# Evaluation of Quality of Life Outcomes Following Palliative Treatment of Bone Metastases with Magnetic Resonance-guided High Intensity Focused Ultrasound: An International Multicentre Study

**DOI:** 10.1016/j.clon.2017.12.023

**Published:** 2018-04

**Authors:** D. Harding, S.L. Giles, M.R.D. Brown, G.R. ter Haar, M. van den Bosch, L.W. Bartels, Y.-S. Kim, M. Deppe, N.M. deSouza

**Affiliations:** ∗ The CRUK Cancer Imaging Centre, The Institute of Cancer Research and Royal Marsden Hospital, MRI Unit, Sutton, Surrey, UK; † Pain Medicine Department, The Royal Marsden Hospital, Sutton, Surrey, UK; ‡ Image Sciences Institute/Imaging Division, University Medical Center Utrecht, The Netherlands; § Department of Radiology and Center for Imaging Science, Samsung Medical Center, School of Medicine, Sungkyunkwan University, Seoul, South Korea; ¶ Department of Radiology, Mint Hospital, Seoul, South Korea; || Philips MR Therapy, Äyritie 4, 01510 Vantaa, Finland

**Keywords:** Bone metastases, cancer-induced bone pain (CIBP), high intensity focused ultrasound (HIFU), magnetic resonance imaging, quality of life (QoL), thermal ablation

## Abstract

**Aims:**

To determine quality of life (QoL) outcomes after palliation of pain from bone metastases using magnetic resonance-guided high intensity focused ultrasound (MR-guided HIFU), measured using the European Organization for Research and Treatment of Cancer (EORTC) QLQ-C15-PAL and the QLQ-BM22 questionnaires.

**Materials and methods:**

Twenty patients undergoing MR-guided HIFU in an international multicentre trial self-completed the QLQ-C15-PAL and QLQ-BM22 questionnaires before and on days 7, 14, 30, 60 and 90 post-treatment. Descriptive statistics were used to represent changes in symptom and functional scales over time and to determine their clinical significance. QoL changes were compared in pain responders and non-responders (who were classified according to change in worst pain score and analgesic intake, between baseline and day 30).

**Results:**

Eighteen patients had analysable QoL data. Clinically significant improvements were seen in the QoL scales of physical functioning, fatigue, appetite loss, nausea and vomiting, constipation and pain in the 53% of patients who were classified as responders at day 30. No significant changes were seen in the 47% of patients who were non-responders at this time point.

**Conclusion:**

Local treatment of pain from bone metastases with MR-guided HIFU, even in the presence of disseminated malignancy, has a substantial positive effect on physical functioning, and improves other symptomatic QoL measures. This indicated a greater response to treatment over and above pain control alone. MR-guided HIFU is non-invasive and should be considered for patients with localised metastatic bone pain and poor QoL.

## Highlights

• High intensity focused ultrasound (HIFU) palliates localised metastatic bone pain.
• Quality of life (QoL) after HIFU improved in pain responders more than in non-responders.
• Systemic QoL improved after HIFU to a single painful site, even in those with disseminated disease.
• Physical functioning was improved after HIFU, even in patients with end-stage disease.

## Introduction

Advances in cancer treatment confer increased survival on patients with bony metastatic disease, but often leave them experiencing chronic metastatic bone pain, which can impact significantly on their quality of life (QoL) [1]. When systemic therapies are inadequate for controlling metastatic bone pain, external beam radiotherapy (EBRT) is offered as a local palliative treatment [2]. This is a well-established and effective treatment that can be delivered non-invasively in an outpatient setting, without immediate side-effects. However, delayed side-effects (including mucositis, fibrosis, gastrointestinal symptoms, fatigue, pathological fractures and neuropathies [3]) can negatively affect patient QoL, even in the 60–80% who experience a pain response [2]. Analysis of the Dutch Bone Metastasis study (*n* = 956, where >70% patients responded to treatment) [4] showed that most QoL domains did not improve after radiotherapy. However, several studies have reported better QoL in radiotherapy responders than non-responders [5], [6], [7]. A literature review of 18 studies [8] concluded that EBRT may provide some improvement or stabilisation in QoL for those who respond to treatment, but did not specify which areas of QoL actually improved.

The non-invasive thermal ablation technique of magnetic resonance-guided high intensity focused ultrasound (MR-guided HIFU) has growing evidence to support its efficacy as a palliative treatment for painful bone metastases [9], [10], [11], [12], [13], with early reports indicating that >70% of patients with radiotherapy refractory metastatic bone pain experienced significant pain reduction within 3 months of HIFU treatment. The largest, phase III study found that 72 of 112 patients (64%) responded to MR-guided HIFU, compared with seven of 35 (20%) reporting a response after a sham treatment [12]. There was a corresponding improvement of 2.4 points (out of 10) in QoL, but this was only briefly summarised using the Brief Pain Inventory Short Form (BPI-SF), a tool that does not differentiate between the multiple factors that influence QoL [14]. As MR-guided HIFU is localised, there is minimal risk of toxicity to normal healthy tissue, potentially conserving a range of QoL measures.

An International consensus panel on clinical trial end points for bone palliation with radiotherapy recommends the QLQ-C15-PAL [15] and the QLQ-BM22 [16] questionnaires as instruments for providing a comprehensive evaluation of QoL [17]. They are validated tools [18], [19], [20] developed by the European Organization for Research and Treatment of Cancer (EORTC). To date, no studies have used these questionnaires to assess QoL after MR-guided HIFU treatment. The purpose of this study, therefore, was to determine the relationship between pain response and specific QoL measures after MR-guided HIFU using both the QLQ-C15-PAL and the QLQ-BM22 questionnaires.

## Materials and Methods

### Study Population

Participants were recruited to an international, prospective, single-arm study, designed to determine the efficacy of MR-guided HIFU for the palliation of painful skeletal metastases (NCT01586273) [21]. Thirty-six patients with bone metastases were assessed, of whom 20 met eligibility criteria (worst pain ≥4/10 on the BPI-SF, corresponding to a bony metastatic site accessible by MR-guided HIFU) and received treatment between May 2012 and July 2016. Recruitment ran across three sites: The Royal Marsden Hospital, Sutton, UK (*n* = 10); University Medical Centre Utrecht, Utrecht, the Netherlands (*n* = 5); and the Samsung Medical Center, Seoul, South Korea (*n* = 5). Patients at all sites provided written informed consent, following approval from an Institutional Review Board (REC number: 12/LO/0424, Samsung Medical Center IRB code: 2013-04-050). The study was conducted in accordance to the principles of the Declaration of Helsinki, Good Clinical Practice and the study protocol. Treatments were carried out using the Philips Sonalleve MR-guided HIFU device. Participants were followed-up on days 7, 14, 30, 60 and 90 days after treatment. All patients included in this QoL analysis had previously received radiotherapy to their painful bone metastases and had experienced differing levels of response. Baseline patient characteristics are presented in Table 1.

**Table 1:** Patient characteristics for *n* = 18 patients who completed the QLQ-C15-PAL and QLQ-BM22 questionnaires at baseline and at least two other follow-up time points

Age (years)	
Mean ± standard deviation	55 ± 11
Median (range)	57 (36–72)
Gender	
Male	9 (50.0%)
Female	9 (50.0%)
Country	
UK	9 (50.0%)
South Korea	5 (27.8%)
Netherlands	4 (22.2%)
Primary tumour site	
Breast	7 (38.9%)
Lung	4 (22.2%)
Liver	4 (22.2%)
Renal	3 (16.7%)
Karnofsky performance status	
Mean ± standard deviation	82 ± 8
Median (range)	80 (70–100)
MR-guided HIFU treatment site	
Pelvis	14 (77.8%)
Arm	2 (11.1%)
Leg	1 (5.6%)
Rib	1 (5.6%)
Prior radiotherapy to target lesion	
8 Gy 1 fraction	2 (11.1%)
20 Gy 5 fractions	3 (16.7%)
30 Gy 10 fractions	4 (22.2%)
High dose >30 Gy, or multiple treatments	9 (50.0%)
Responder to prior radiotherapy? (complete or partial)	7 (38.9%)
Number of months radiotherapy to HIFU screening: median (range)	5 (1–57)
Number of painful sites (*n* = 16)	
Mean ± standard deviation	2 ± 1
Median (range)	2 (1–6)
NRS worst pain score	
Mean ± standard deviation	7 ± 1
Median (range)	7 (3–9)
MEDD∗ (mg)	
Median (range)	10 (0–1000)†

MR-guided HIFU, magnetic resonance-guided high intensity focused ultrasound; MEDD, morphine equivalent daily dose; NRS, numerical rating scale.

∗ The conversion ratios for calculating MEDD were as follows: 1 mg oral oxycodone = 2 mg oral morphine, 1 μg/h fentanyl patch = 3.3 mg oral morphine, 12 mg oral codeine = 1 mg oral morphine.

† In one case, the calculated MEDD was >1000 mg, but conversion is inaccurate at these doses, and so the value was censored to 1000 mg.

### Questionnaires

Assessment of baseline QoL occurred on the day of MR-guided HIFU treatment before treatment was administered. A further QoL assessment was completed at each follow-up time point. The QoL questionnaires were self-completed by patients during their hospital visits at baseline, 30, 60 and 90 days, and at home at the 7 and 14 day time points.

The QLQ-C15-PAL [15] is a shortened version of the EORTC QLQ-C30 [22] and contains 15 items. It was developed as an abbreviated tool to assess QoL in patients treated palliatively. Data collection in advanced cancer patients is facilitated by reducing the burden of completing the longer, more time-consuming QLQ-C30. The QLQ-C15-PAL contains seven symptom scales (dyspnoea, pain, insomnia, fatigue, appetite loss, nausea and vomiting, and constipation) and three functional scales (physical functioning, emotional functioning, and overall QoL), which were identified as being relevant to the palliative population.

The QLQ-BM22 [16] was developed as a specific module for assessing QoL in patients with bone metastases. It is a 22-item questionnaire comprising two multi-item symptom scales: painful sites (five items) and painful characteristics (three items) and two multi-item functional scales: functional interference (eight items) and psychosocial aspects (six items).

Items on both the QLQ-C15-PAL and QLQ-BM22 questionnaires were rated on a four-point Likert scale and were rated from 1 (not at all) to 4 (very much), with the exception of the overall QoL status item, which was rated from 1 (very poor) to 7 (excellent). A higher score for the symptom scales represents a higher level of symptomatology and, therefore, a decreased QoL. By contrast, a higher score for the functional scales represents a higher level of functionality and, therefore, an increased QoL. Each scale was transformed to a score ranging from 0 to 100, according to their respective scoring manual.

### Magnetic Resonance-guided High Intensity Focused Ultrasound Response Classification

Patients were categorised as responders to MR-guided HIFU treatment if they experienced a complete response or a partial response at day 30 after treatment, and non-responders if they experienced no response or pain progression at day 30. This was determined using the international consensus for clinical trial end points on bone pain palliation with radiotherapy criteria [17] whereby a complete response was defined by a BPI-SF worst pain score of zero, with no concomitant increase in analgesic intake. A partial response was defined as a reduction of ≥2 (0–10 scale) in worst pain score, without analgesic increase; or analgesic reduction of ≥25%, without an increase in worse pain score. Similarly, pain progression was defined as an increase of ≥2 in worst pain score, without analgesic decrease; or analgesic increase of ≥25%, with the worst pain score stable or 1 point above baseline. No response applied to all other cases. The ≥ or <25% change in analgesia was determined by calculating the change in morphine equivalent daily dose [23]; for non-opioid medication where morphine equivalent daily dose cannot be calculated, the magnitude of reductions in dose were established through comparison with baseline dose.

### Statistical Analysis

All analyses were conducted on GraphPad Prism (version 7.00 for Windows, GraphPad Software, La Jolla, California, USA; www.graphpad.com).

Descriptive statistics, such as mean, median and standard deviation for continuous values, and counts and proportions for categorical values, were used. Normality tests, including skewness, kurtosis and the D'Agostino-Pearson test, were conducted for each symptom and functional scale at baseline. Descriptive statistics were used to represent the changes in QoL over time.

Pearson's correlation between mean pain score as measured by the BPI-SF and mean pain score as measured by the QLQ-C15-PAL at baseline and day 30 was conducted to ensure the pain level was reported consistently across measures.

Independent sample *t*-tests were used to identify any significant differences between responders and non-responders in each scale at baseline. For all statistical tests, *P* values were considered to indicate significance below the 0.05 level.

Minimal clinically important differences (MCIDs) [24] were compared against the mean change scores from baseline at each time point and were used as thresholds for clinically significant improvement or deterioration. The published MCIDs were decided based on a population of patients completing the QLQ-C15-PAL and the QLQ-BM22 in relation to receiving palliative radiotherapy for painful bone metastases.

## Results

### Data Completeness and Response Classification

Eighteen patients completed the QoL assessment at baseline and at least two other follow-up time points at days 7, 14 or 30, and so were included in this QoL analysis. Two patients were excluded as they withdrew from the study before day 30 due to progression of underlying disease. Multi-item scales were included in analyses if at least 50% of the items included in the scale were answered. One patient who had not completed the QLQ-BM22 at day 14 completed all other questionnaires at all other time points. Additionally, another patient had not completed either questionnaire at day 30. Therefore, the number of participants at the day 30 follow-up was *n* = 17. The same patient did not complete the BPI-SF at the day 30 time point and therefore their response to treatment could not be assessed. One patient had not completed the psychosocial aspects scale from the QLQ-BM22 at day 14, and another patient had not completed the second side of the QLQ-C15-PAL questionnaire at baseline and so their data were not available for the constipation, fatigue, emotional functioning and overall QoL scales at this time point, and was not included in the determination of clinical significance.

The number of patients remaining in the study decreased to *n* = 13 at the day 60 follow-up and *n* = 11 at the day 90 follow-up.

Pain response rates (both complete and partial) to MR-guided HIFU at days 7, 14, 30, 60 and 90 were 38.9% (*n* = 18), 61.1% (*n* = 18), 53% (*n* = 17), 61.5% (*n* = 13) and 63.6% (*n* = 11), respectively. There was a significant, positive correlation between the QLQ-C15-PAL pain symptom scale and the BPI-SF worst pain score at baseline (*r* = 0.61, *P* = 0.008) and day 30 (*r* = 0.62, *P* = 0.008), meaning pain was reported consistently across the two measures.

Six serious adverse events were reported in four of 18 patients and all were classed as being unrelated to both the study and the treatment device.

### Whole Cohort Quality of Life

Mean ± standard deviation scores for each symptom and functional scale over time are presented in Table 2. All scales were normally distributed, with the exception of the dyspnoea and nausea and vomiting scales.

**Table 2:** Overall mean ± standard deviation scores for quality of life scales for 2 validated quality of life questionnaires: QLQ-C15-PAL and QLQ-BM22

	Baseline	Day 7	Day 14	Day 30	Day 60	Day 90
QLQ-C15-PAL						
Physical functioning†	57.77 ± 24.4	64.43 ± 22.27	66.66 ± 21.18	59.21 ± 30.61	65.12 ± 26.67	72.1 ± 25.08
Emotional functioning†	69.62 ± 22.6	65.76 ± 17.11	63.44 ± 22.52	72.56 ± 18.33	73.08 ± 22.85	72.74 ± 24.73
Dyspnoea∗	7.41 ± 18.28	14.81 ± 17.04	14.81 ± 20.52	9.803 ± 15.65	15.38 ± 17.29	9.09 ± 15.57
Pain∗	66.67 ± 17.15	59.26 ± 20.79	54.63 ± 19.64	54.9 ± 22.64	46.15 ± 29.78	40.91 ± 28.25
Insomnia∗	38.89 ± 36.6	31.48 ± 26.75	31.48 ± 24.18	31.37 ± 32.21	25.64 ± 24.17	21.21 ± 16.82
Fatigue∗	50.32 ± 29.71	47.53 ± 19.75	38.26 ± 21.65	47.06 ± 22.43	36.75 ± 22.42	37.36 ± 17.43
Appetite loss∗	35.19 ± 35.19	22.22 ± 32.34	16.67 ± 26.2	29.41 ± 33.09	12.82 ± 21.68	9.091 ± 21.56
Nausea and vomiting∗	15.76 ± 24.57	12.05 ± 15.98	8.34 ± 13.11	15.69 ± 23.18	5.14 ± 8.02	0 ± 0
Constipation∗	29.41 ± 30.92	31.48 ± 31.25	27.78 ± 32.84	17.65 ± 29.15	12.82 ± 28.99	21.21 ± 34.23
Overall quality of life†	49.99 ± 22.04	51.85 ± 24.17	58.33 ± 22.31	50.98 ± 24.62	56.41 ± 25.02	56.07 ± 27.14

QLQ-BM22						
Painful sites∗	40 ± 18.58	37.53 ± 19.25	30.2 ± 15.83	31.76 ± 15.19	32.31 ± 16.29	34.55 ± 21.04
Painful characteristics∗	43.83 ± 20.34	41.05 ± 17.79	39.21 ± 16.25	38.56 ± 18.48	33.33 ± 20.29	31.31 ± 15.57
Functional interference†	45.93 ± 18.08	55.09 ± 14.94	58.26 ± 15.92	52.24 ± 24.64	59.8 ± 27.53	63.15 ± 23.29
Psychosocial aspects†	54.63 ± 18.79	59.57 ± 16.59	57.64 ± 15.96	52.94 ± 25.09	55.56 ± 17.57	55.05 ± 18.17

∗ High scores represent high symptomatology/decreased quality of life.

† High scores represent high functionality/increased quality of life.

**Fig 1:**
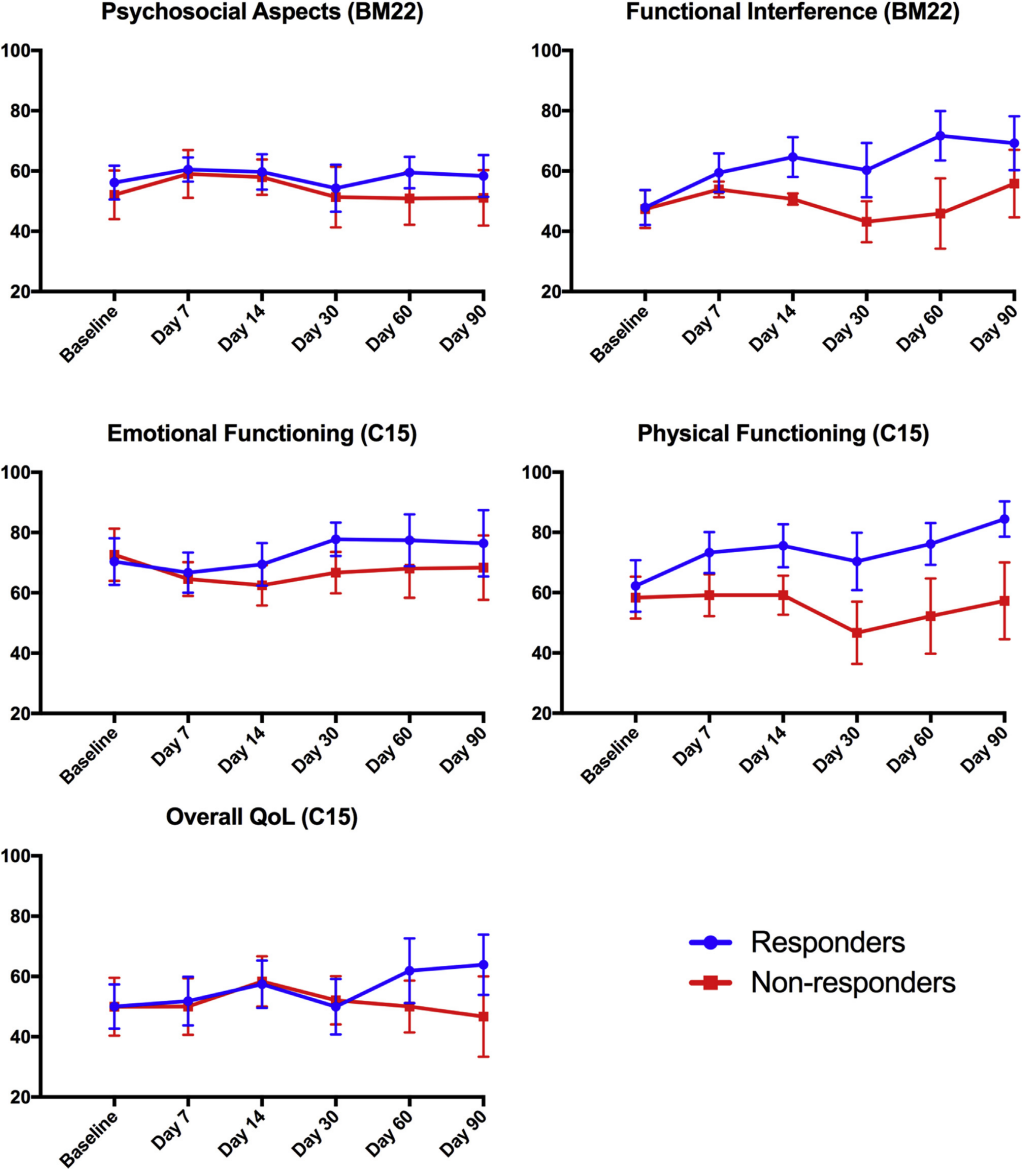
Graphs representing changes between responders and non-responders in each QLQ-BM22 and QLQ-C15-PAL functional scale over time. The vertical axis is the mean score and the horizontal axis is the study time point. Error bars show standard error of the mean (SEM). Higher scores represent high functionality, i.e. increased quality of life.

**Fig 2:**
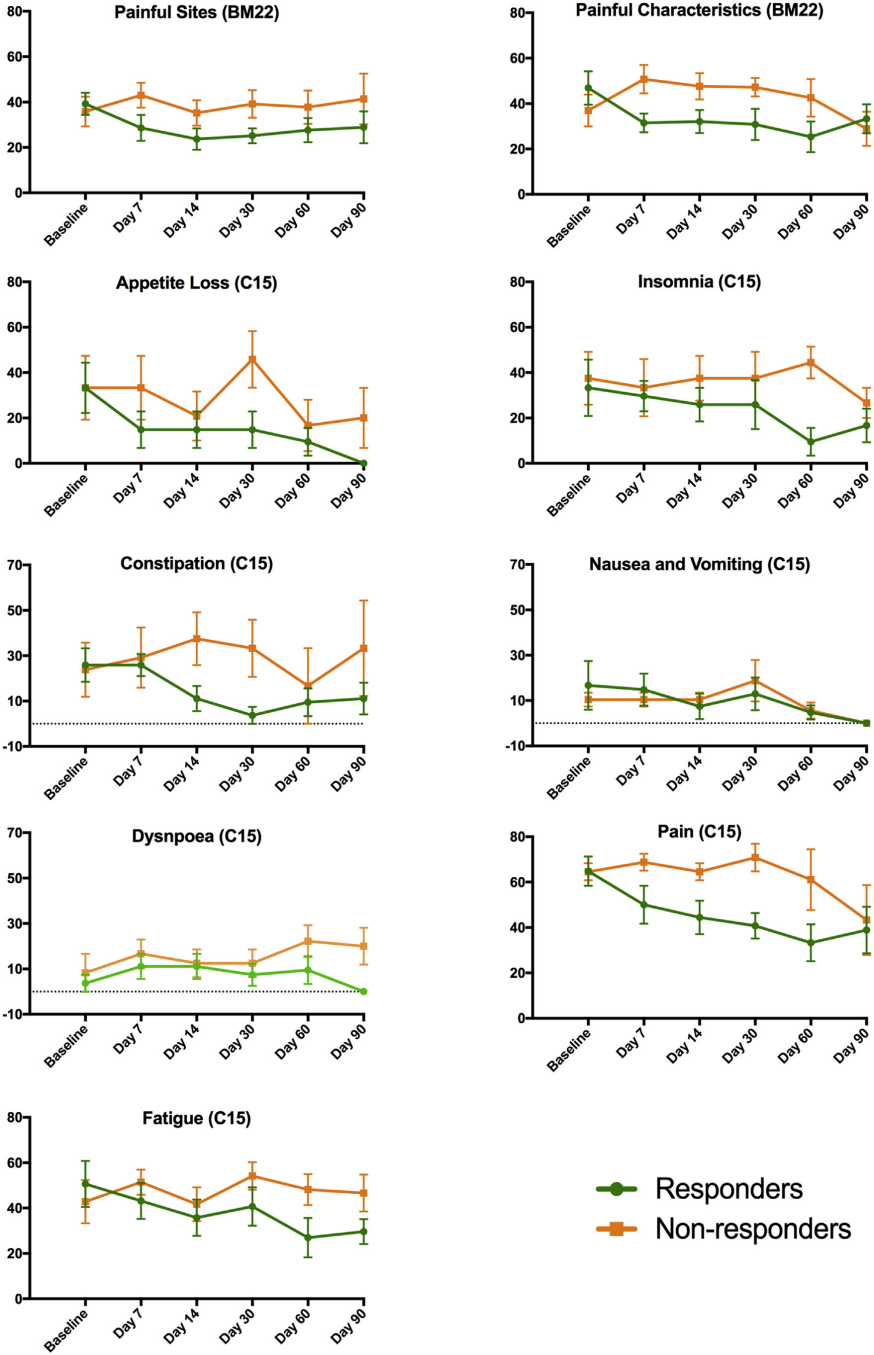
Graphs representing changes between responders and non-responders in each QLQ-BM22 and QLQ-C15-PAL symptom scale over time. The vertical axis is the mean score and the horizontal axis is the study time point. Error bars show standard error of the mean (SEM). Lower scores represent diminished symptoms, i.e. increased quality of life.

No significant differences were found between responders' and non-responders’ baseline scores in any symptom scales. The baselines scores, and the trajectory of each QoL scale over time, are presented in Fig 1, Fig 2. Figure 1 displays the functioning scales from both the QLQ-C15-PAL and the QLQ-BM22, for which an increase in scores represents high functionality and improved QoL; Figure 2 displays the symptom scales, for which an increase in scores represents high symptomatology and decreased QoL.

### Quality of Life in Responders to Magnetic Resonance-guided High Intensity Focused Ultrasound

Patients classed as responders at day 30 experienced a mean 18% increase in physical functioning at day 7. This steadily increased to 36% by day 90, with the exception of a small dip at day 30 to 13%. The mean scores for insomnia, fatigue, appetite loss, nausea and vomiting, and constipation all reduced by ≥14 points by study end, equating to a 50%, 41%, 100%, 100% and 57% reduction in symptoms, respectively. Dyspnoea and emotional functioning remained relatively consistent across time points. Improvements in overall QoL were distinguishable by day 60, when there was a 24% improvement in scores, which increased to 28% at study end. This was in the context of an improvement in pain, as measured by the QLQ-C15-PAL, by up to 49%. There were also improvements in the painful sites scale (>26%) and the painful characteristics scale (>29%) in responders. The functional interference scale of QLQ-BM22 followed a similar pattern to the physical functioning scale from the QLQ-C15-PAL by showing a persistent and gradually increasing improvement across time points, with a slight dip in scores at day 30. Psychosocial aspect scores remained relatively stable throughout the study.

### Quality of Life in Non-responders to Magnetic Resonance-guided High Intensity Focused Ultrasound

The QLQ-C15-PAL showed that non-responders had little improvement in physical functioning across any of the time points, and a worsening of physical functioning at day 30 by 20%. The mean scores for the insomnia, appetite loss and nausea and vomiting scales had improved by ≥10 points by the study end, equating to a 29%, 40% and 100% reduction in symptoms, respectively. However, scores were consistently worse than those of the responders at each follow-up time point, with the exception of the nausea and vomiting response scale at day 7. The fatigue scale remained relatively constant throughout the study, with a small worsening of 9% by day 90. There was also a substantial (40%) worsening in constipation scores at study end. Emotional functioning saw a 6% worsening in scores by day 90, compared with a 9% improvement seen in responders. In terms of overall QoL there were no notable changes, with a small decrease (7%) by study end. Although pain scores remained relatively constant for non-responders up to day 60, there was an improvement at day 90 by 33%. Using the QLQ-BM22, non-responders saw a 15% worsening in painful sites scores by day 90. The 22% improvement in painful characteristics scores at study end was preceded by a consistent worsening of scores by an average of 27%. The functional interference and psychosocial aspects scales remained relatively constant throughout, reflecting similar trends as in the physical and emotional functioning scales seen in the QLQ-C15-PAL for non-responders.

### Comparison against Minimal Clinically Important Differences

Table 3 compares the QoL change scores in responders versus non-responders with the distribution-based MCIDs [24]. Clinically significant improvements were seen in responders in the fatigue, appetite loss, nausea, constipation and physical functioning scales of the QLQ-C15-PAL, and within the painful sites, painful characteristics and functional interference scales of the QLQ-BM22. Responders had no significant deterioration on any scale. Non-responders saw no clinically significant improvements in QoL scales, with the exception of the pain scale at day 90, and saw clinically significant deterioration in the physical functioning and painful characteristics scales at one time point each.

**Table 3:** Mean change scores compared with baseline

	Responders					Non-responders				
	Day 7	Day 14	Day 30	Day 60	Day 90	Day 7	Day 14	Day 30	Day 60	Day 90
QLQ-C15-PAL										
Fatigue∗	–7.43	**–14.84**‡	–9.90	**–23.65**‡	**–21.01**‡	8.56	–1.18	11.34	5.31	3.82
Appetite loss∗	**–18.52**‡	**–18.52**‡	**–18.52**‡	**–23.81**‡	**–33.33**‡	0.00	–12.5	12.50	–16.67	–13.33
Nausea and vomiting∗	–1.86	–9.27	–3.71	–11.91	–16.68 ‡	0.00	0.00	8.31	–4.87	–10.44
Constipation∗	0.00	–14.81	**–22.22**‡	–16.40	–14.81	5.36	13.69	9.52	–7.14	9.52
Pain∗	**–14.82**‡	**–20.37**‡	**–24.07**‡	**–31.48**‡	**–25.93**‡	4.17	0.00	6.25	–3.47	**–21.25**‡
Emotional functioning†	–3.70	–0.92	7.40	7.02	6.02	–8.03	–10.10	–5.94	–4.58	–4.29
Physical functioning†	11.11	**13.33**‡	8.14	**13.96**‡	**22.21**‡	0.81	0.83	**–11.66**§	–6.12	–1.02
QLQ-BM22										
Painful sites∗	**–10.62**‡	**–15.56**‡	**–14.07**‡	**–11.64**‡	**–10.37**‡	7.22	–0.60	3.33	1.94	5.50
Painful characteristics∗	**–15.43**‡	**–14.82**‡	**–16.05**‡	**–21.52**‡	**–13.58**‡	**13.82**§	10.64	10.24	5.61	–8.09
Functional interference†	**11.57**‡	**16.73**‡	**12.43**‡	**23.80**‡	**21.36**‡	6.51	3.28	–4.24	–1.46	8.44
Psychosocial aspects†	4.32	3.55	–1.85	3.35	2.16	6.94	5.85	–0.70	–1.16	–0.97

Scores above the minimal clinically important differences (MCIDs) set by Raman *et al.*[24] are highlighted in bold. No MCIDs available for the dyspnoea, insomnia and overall quality of life scales.

∗ Increasing scores represent worsening quality of life.

† Increasing scores represent improving quality of life.

‡ Clinically significant improvement.

§ Clinically significant deterioration.

## Discussion

Our study shows that beneficial QoL changes occur after MR-guided HIFU treatment, in addition to improvements in pain, which indicate even greater responses to treatment than when assessed using pain criteria alone. Physical function and symptoms, including appetite loss, fatigue, insomnia and constipation, were improved across the whole cohort, reflecting the importance of monitoring the individual elements that constitute QoL when considering patient outcome. With an increasing number of cancer survivors, the control of metastatic bone pain is critical in maintaining QoL, making interventions that achieve this (such as MR-guided HIFU) of paramount importance.

The reduction in pain scores measured by the BPI-SF and the QLQ-C15-PAL were associated with an increase in physical functionality as assessed by the QLQ-C15-PAL, and further corroborated by the functional interference scale in the QLQ-BM22. Improved physical functioning may improve the patients' ability to complete activities that are a part of daily life. The slight worsening of scores in the physical functioning scale for both responders and non-responders at day 30 (compared with later post-treatment time points) was unexpected. These patterns remained even when accounting for patients who withdrew from the study before day 90. It is hard to draw firm conclusions on the reason for this from our small cohort. However, one theory relates to bone weakness, which, pre-clinical studies have shown to occur after MR-guided HIFU, predominantly 4–6 weeks after treatment, returning to normal levels at 3 months [25], [26]. As bone weakness is known to have a significant effect on physical functioning [27] this could be a possible explanation. Fortunately, no post-treatment fractures occurred in our group, although this remains a theoretical risk.

Although the clinically significant reductions in fatigue, appetite loss and nausea and vomiting may be as a direct result of improvement in pain scores, there could also be an indirect effect through a reduction in analgesic intake as a result of reduced pain. Also, although the initial low score of ∼26 for constipation may be subject to floor effects, the mean changes show responders consistently experienced at least a 57% improvement in constipation symptoms from day 14 onwards, indicating that a reduction in opioid analgesic consumption caused this improvement [28], [29]. This feature has been reported previously as significantly reducing QoL [30]. Conversely, non-responders experienced no clinically significant improvements for any of the symptoms at any of the time points. Dyspnoea remained unaffected, as it is not associated with bone metastases. Even in the presence of concurrent pulmonary metastases, treatment of bone pain would not be expected to affect dyspnoea.

Interestingly both the emotional functioning and psychosocial aspects scales remained relatively stable, suggesting that improvement in other aspects of QoL was insufficient to constitute an improvement in emotional functioning. This is to be expected in end-stage patients with extensive metastatic disease. In fact, the end-stage nature of this cohort needs to be considered; 17% of patients died during the study. It is therefore reassuring that at a time when QoL would usually be worsening [4], [31], it is possible with a non-invasive single treatment to improve overall QoL for many patients within a 3 month time frame, by reducing pain and analgesic use and thus improving physical functioning to allow self-care activities.

The response for the purposes of this QoL assessment was based on classification at day 30, as this was determined *a priori* as the primary end point for measuring pain response. However, this did not reflect the changing responses some patients experienced throughout the study. In fact, 27% of patients who remained in the study until day 90 would have been classified differently then, compared with their classification at day 30. This may explain why the pain scale of the QLQ-C15-PAL and the painful characteristics scale of the QLQ-BM22 decreased to a similar level in responders and non-responders by the study end. In our small cohort, the assessment of pain and QoL may have been better classified later than 30 days after treatment. Although this observation might not be generalisable, it does indicate a need for longer-term follow-up. In our experience, the duration of improvements in pain and QoL in all participants after a single treatment was highly encouraging.

As in some other studies [12], all our patients had received previous EBRT to their painful bone metastasis and, therefore, it was not possible to compare the effect on QoL between patients with and without previous EBRT treatment. A QoL assessment has not been conducted previously in radiation naive patients, in whom pain response rates to HIFU of 89% (with 72% of patients experiencing complete pain relief) have been reported [32]. These pain response rates are noticeably higher than in EBRT refractory patients, indicating that MR-guided HIFU may well have an even greater impact on QoL if given as an initial palliative option. However, as EBRT is the current standard of care for patients referred with localised bone pain, it would be beneficial to directly compare QoL outcomes in patients receiving EBRT and MR-guided HIFU for metastatic bone pain, to determine which method has the greatest impact on QoL. Although our study can be compared with those using the same QoL measures to examine the impact of EBRT on QoL [33], variations in trial methodologies make comparison of the two treatments difficult. A phase III randomised controlled trial (NCT01091883) [34] is currently underway that aims to assess safety, pain and QoL outcomes in patients receiving MR-guided HIFU or EBRT. Greater understanding of any pain and QoL differences after MR-guided HIFU or EBRT will be helpful in determining the optimal treatment pathways for patients with localised painful bone metastases.

By using the QLQ-C15-PAL, a palliative-specific questionnaire, we have been able to determine a variety of symptom and functional changes that can be easily interpreted from a clinical perspective. In addition, using the QLQ-BM22, a bone metastasis-specific questionnaire, we have been able to reliably assess a range of QoL issues that are the most pertinent for patients with bone metastases. However, both questionnaires have limitations. Neither questionnaire measures spiritual components, which have been found to be equally important to physical symptoms for QoL [35]. Although it could be beneficial to add further questions assessing this issue, this would risk placing an additional burden on patients. The small sample size is another limitation of this study and the large standard deviations made it inappropriate to carry out relevant statistical tests. This is a particular issue for subjective and qualitative assessments such as QoL and future studies would benefit from larger sample sizes. However, evaluation of clinical significance through MCIDs provides evidence for the improvements in QoL seen among responders.

## Conclusion

Our data show that patients with disseminated malignancy and focal metastatic bone pain, refractory to palliative radiotherapy, can experience substantial QoL improvements after a single MR-guided HIFU treatment. We observed improvements in both pain and physical functioning, which may have been related to decreased reliance on analgesics, with subsequent reduction in their side-effects. We did not observe any treatment-related serious adverse effects. Further studies that will evaluate QoL outcomes in treatment-naïve patients undergoing either EBRT or MR-guided HIFU will be important in determining the optimal treatment pathways for patients with painful bone metastases.
